# Prevalence of pathogenic germline variants in the circulating tumor DNA testing

**DOI:** 10.1007/s10147-022-02220-x

**Published:** 2022-07-23

**Authors:** Yoshihiro Yamamoto, Keita Fukuyama, Masashi Kanai, Tomohiro Kondo, Masahiro Yoshioka, Tadayuki Kou, Pham Nguyen Quy, Reiko Kimura-Tsuchiya, Takahiro Yamada, Shigemi Matsumoto, Shinji Kosugi, Manabu Muto

**Affiliations:** 1 Department of Therapeutic Oncology, Graduate School of Medicine, Kyoto University, 54 Kawahara-cho, Shogoin, Sakyo-ku, Kyoto, 606-8507 Japan; 2 Clinical Genetics Unit, Kyoto University Hospital, 54 Kawahara-cho, Shogoin, Sakyo-ku, Kyoto, 606-8507 Japan; 3 Department of Medical Ethics and Medical Genetics, Kyoto University School of Public Health, Yoshida-Konoe-cho, Sakyo-ku, Kyoto, 606-8501 Japan

**Keywords:** Circulating tumor DNA testing, Presumed germline pathogenic variants, Variant allele fractions, Confirmatory germline sequencing

## Abstract

**Background:**

Somatic and germline variants are not distinguishable by circulating tumor DNA (ctDNA) testing without analyzing non-tumor samples. Although confirmatory germline testing is clinically relevant, the criteria for selecting presumed germline variants have not been established in ctDNA testing. In the present study, we aimed to evaluate the prevalence of pathogenic germline variants in clinical ctDNA testing through their variant allele fractions (VAFs).

**Methods:**

A total of consecutive 106 patients with advanced solid tumors who underwent ctDNA testing (Guardant360^®^) between January 2018 and March 2020 were eligible for this study. To verify the origin of pathogenic variants reported in ctDNA testing, germline sequencing was performed using peripheral blood DNA samples archived in the Clinical Bioresource Center in Kyoto University Hospital (Kyoto, Japan) under clinical research settings.

**Results:**

Among 223 pathogenic variants reported in ctDNA testing, the median VAF was 0.9% (0.02–81.8%), and 88 variants with ≥ 1% VAFs were analyzed in germline sequencing. Among 25 variants with ≥ 30% VAFs, seven were found in peripheral blood DNA (*BRCA2*: *n* = 6, *JAK2*: *n* = 1). In contrast, among the 63 variants with VAFs ranging from 1 to < 30%, only one variant was found in peripheral blood DNA (*TP53*: *n* = 1). Eventually, this variant with 15.6% VAF was defined to be an acquired variant, because its allelic distribution did not completely link to those of neighboring germline polymorphisms.

**Conclusion:**

Our current study demonstrated that VAFs values are helpful for selecting presumed germline variants in clinical ctDNA testing.

**Supplementary Information:**

The online version contains supplementary material available at 10.1007/s10147-022-02220-x.

## Introduction

Next-generation sequencing (NGS)-based circulating tumor DNA (ctDNA) testing is an alternative method for comprehensive genomic profiling. This method is widely used in the clinical practice of cancer treatment because of its minimal invasiveness and no requirement for tissues. In ctDNA testing, small fractions of ctDNA can be detected among the total cell-free DNAs by incorporating a combination of molecular barcoding technology and bioinformatics methods [1]. Previous studies reported that the variant allele fractions (VAFs) of ctDNA range widely from 0.1 to ≥ 90%, although the median VAFs are less than 1% [2, 3]. VAFs reported in ctDNA testing are affected by several clinical variables, including the cancer type, stage, and overall tumor load [4–7].

Comprehensive genomic profiling sometimes results in secondary germline findings (SFs). The American College of Medical Genetics and Genomics (ACMG) recommended reporting SFs for several genes responsible for hereditary diseases [8–10]. However, in tumor-only testing and ctDNA testing, confirmatory germline testing is required, which uses non-tumor samples such as peripheral blood DNA. The European Society of Medical Oncology Precision Medicine Working Group recommended a threshold of ≥ 30% (or ≥ 20% for small insertions–deletions) for VAFs as an indication of presumed germline pathogenic variants in tumor-only tissue sequencing [11]. These recommendations are now widely used for managing SFs in tumor-only tissue sequencing in clinical practice. However, if the same threshold of ≥ 30% VAFs is feasible in clinical ctDNA testing is not well understood. Previous studies suggested that germline variants were presumable based on higher VAFs in ctDNA testing [12, 13], but the studies verifying this idea by germline testing are still limited.

In this present study, we performed germline sequencing to investigate the prevalence of true germline variants using peripheral blood DNA samples archived in the Clinical Bioresource Center in Kyoto University Hospital under clinical research settings.

## Patients and methods

### Patients

Consecutive 106 patients with unresectable advanced cancers who underwent Guardant360^®^ liquid biopsy (Guardant Health, Redwood City, CA) in Kyoto University Hospital between January 2018 and March 2020 were eligible. Studies were approved by the Ethics Committee of the Kyoto University Graduate School of Medicine (Kyoto, Japan; G692 and G1223). All participants provided written informed consent to donate their blood samples to Clinical Bioresource Center in Kyoto University Hospital (Kyoto, Japan) for research use.

### Confirmatory germline sequencing

According to the established recommendations for SFs [8, 9, 14], we selected 18 genes associated with hereditary cancer syndromes targeted by Guardant360^®^ and designed a custom amplicon sequencing panel covering these 18 genes (Ion AmpliSeq On-Demand Panel, Thermo Fisher Scientific, MA, USA) (Supplementary Table S1). This panel covered the coding sequences and the splice sites of target genes. In addition, we also used the Ion AmpliSeq Cancer Hotspot Panel v2 (Thermo Fisher Scientific), which covered common pathogenic mutations in additional 29 cancer-related genes targeted by Guardant360^*®*^ (Supplementary Table S1). Germline sequencing was performed in clinical research settings regardless of clinical indication.

Peripheral blood DNA was extracted using the Gene Prep Star NA-480 system (Kurabo Industries, Osaka, Japan) and archived in Clinical Bioresource Center in Kyoto University Hospital. To prepare library samples, the DNA was processed using the Ion AmpliSeq Library Kit Plus (Thermo Fisher Scientific). It was analyzed using an Ion S5 sequencing system equipped with Torrent Suite 5.10.1 (Thermo Fisher Scientific). Variants identified by Torrent Suite variantCaller were annotated using SnpEff tools. To avoid false-positive mutation calls, variant reads less than ten or VAFs less than 1.0% were excluded. Variants detected with VAFs between 1 and 30% in peripheral blood DNA were suspected to be somatically acquired variants that possibly include clonal hematopoiesis (CH) and other genetic mosaicisms rather than inherited germline variants [15, 16], and further information regarding their copy number and allelic distribution were obtained as follows. Copy-number alterations were analyzed using CovCopCan software (v.1.3.3) [17] to evaluate possible effects on VAFs. In addition, the distribution of variant allele was compared with those of heterozygous common single-nucleotide polymorphisms (SNPs) on the same sequencing reads using the Integrative Genomics Viewer (Broad Institute, MA, USA). Acquired variants were defined if their allelic distribution did not completely link to those of neighboring SNPs.

### Definition of variant pathogenicity

To define variant pathogenicity, we first referred to ClinVar (https://www.ncbi.nlm.nih.gov/clinvar/). If variants were registered as “pathogenic” or “likely-pathogenic” in ClinVar, they were classified into pathogenic variants. The variants registered as “conflicting interpretations of pathogenicity” were also classified as pathogenic if they demonstrated at least one pathogenic or likely-pathogenic report in ClinVar. For frameshift or truncation variants with no registration in ClinVar, their pathogenicity was considered by the annotations of neighboring truncated variants. If variants exhibited no information in ClinVar, their pathogenicity was evaluated based on the ACMG and the Association for Molecular Pathology guidelines [18].

## Results

### Patient characteristics

The characteristics of patients who underwent ctDNA testing are summarized in Table 1. The patients’ median age was 64 years (28–82), and 57 patients (53.8%) were male. The most common type of cancer was pancreatic (*n* = 37), followed by colorectal (*n* = 12), and lung (*n* = 11). Twenty patients (18.9%) presented with a family history of cancer in both first-degree and second-degree relatives.

**Table 1:** Characteristics of the patients who underwent ctDNA testing

Characteristics	No. of patients (%)		
	ctDNA tested *n* = 106		Germline tested *n* = 44
Age, years			
Median [range]	64 [28–82]		65 [38–80]
Sex			
Male	57 (53.8)		26 (59.1)
Female	49 (46.2)		18 (40.9)
Cancer type			
Pancreatic	37 (34.9)		19 (43.2)
Colorectal	12 (11.3)		7 (15.9)
Lung	11 (10.4)		5 (11.4)
Breast	10 (9.4)		3 (6.8)
Bile duct	9 (8.5)		2 (4.5)
Ovarian	4 (3.8)		3 (6.8)
Prostate	3 (2.8)		2 (4.5)
Esophageal	3 (2.8)		1 (2.3)
Head and neck	3 (2.8)		0 (0)
Urothelial	2 (1.9)		0 (0)
Gastric	2 (1.9)		2 (4.5)
Cervical	2 (1.9)		0 (0)
Endometrial	1 (0.9)		0 (0)
Small intestinal	1 (0.9)		0 (0)
Hepatic	1 (0.9)		0 (0)
Thyroid	1 (0.9)		0 (0)
NEC	1 (0.9)		0 (0)
Other	3 (2.8)		0 (0)
Familial history of any cancers			
Both in FDR/SDR	20 (18.9)		8 (18.2)
Only in FDR	43 (40.6)		21 (47.7)
Only in SDR	17 (16.0)		5 (11.4)

*VAFs* variant allele fractions, *FDR* first-degree relatives, *SDR* second-degree relatives

### Distribution of pathogenic variant allele fractions in ctDNA testing

**Fig. 1:**
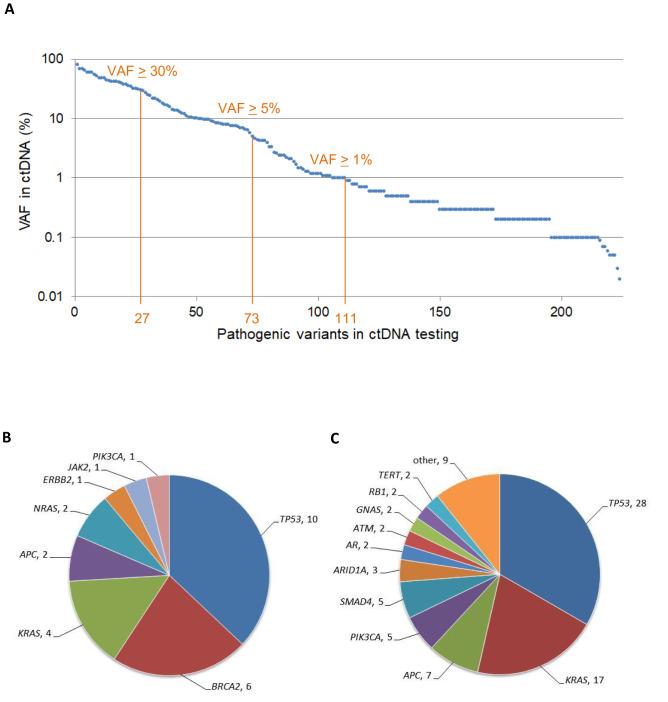
**A** The distribution of variant allele fractions of pathogenic variants found in ctDNA testing. Two hundred and twenty-four pathogenic variants detected in ctDNA testing are arranged by their variant allele fractions (VAFs) from highest to lowest. **B** The number of pathogenic variants with 30% or higher VAFs arranged by gene in ctDNA testing (*n* = 27). **C** The number of pathogenic variants with VAFs ranged from 1 to < 30% by gene in ctDNA testing (*n* = 84). Other includes one variant in *BRAF*, *BRCA2*, *CDKN2A*, *CCNB1*, *ERBB2*, *FBXW7*, *FGFR2*, *NFE2L2,* and *PTEN*

In total, 409 short variants were reported in 106 patients, and 224 of those variants were classified as pathogenic. The median VAF of pathogenic variants was 0.9% (0.02–81.8%). The number of pathogenic variants with ≥ 1%, ≥ 5%, and ≥ 30% VAFs was 111, 73, and 27, respectively (Fig. 1A). Among 27 variants with ≥ 30% VAFs (high VAFs), *TP53* variants were the most common (*n* = 10), followed by *BRCA2* (*n* = 6), and then *KRAS* (*n* = 4) (Fig. 1B). Similarly, among 84 pathogenic variants with moderate VAFs ranging from 1 to < 30%, *TP53* variants were most common (*n* = 28), followed by *KRAS* (*n* = 17), *APC* (*n* = 7), *SMAD4* (*n* = 5), and *PIK3CA* (*n* = 5) (Fig. 1C).

### Results of germline sequencing

**Fig. 2:**
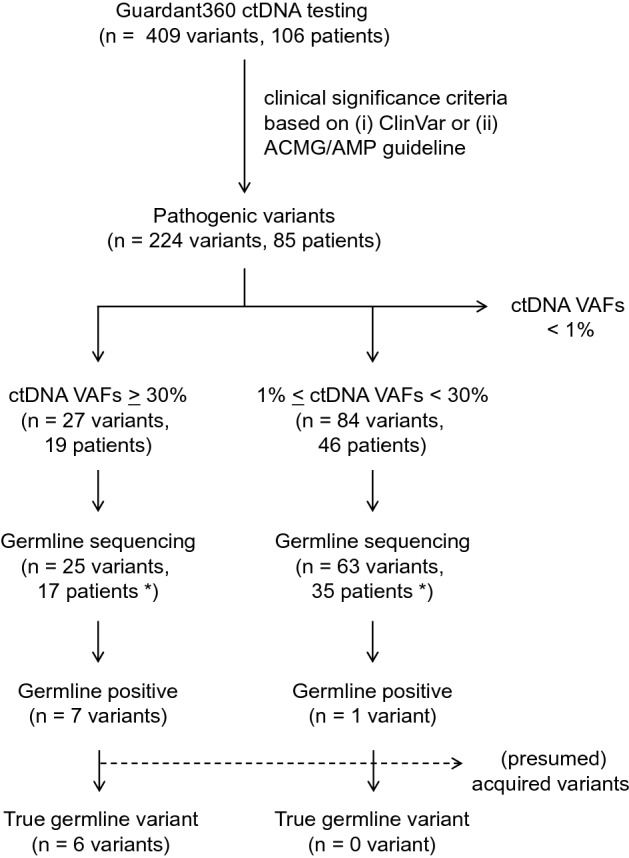
Flow chart of the methods in the study showing the downstream flow for germline sequencing and results of ctDNA testing.(*) Since the data of eightpatients with VAFs >30% overlapped those with VAFs ranging from 1% to 30%, the total number of patients who underwent germline sequencing was 44

**Fig. 3:**
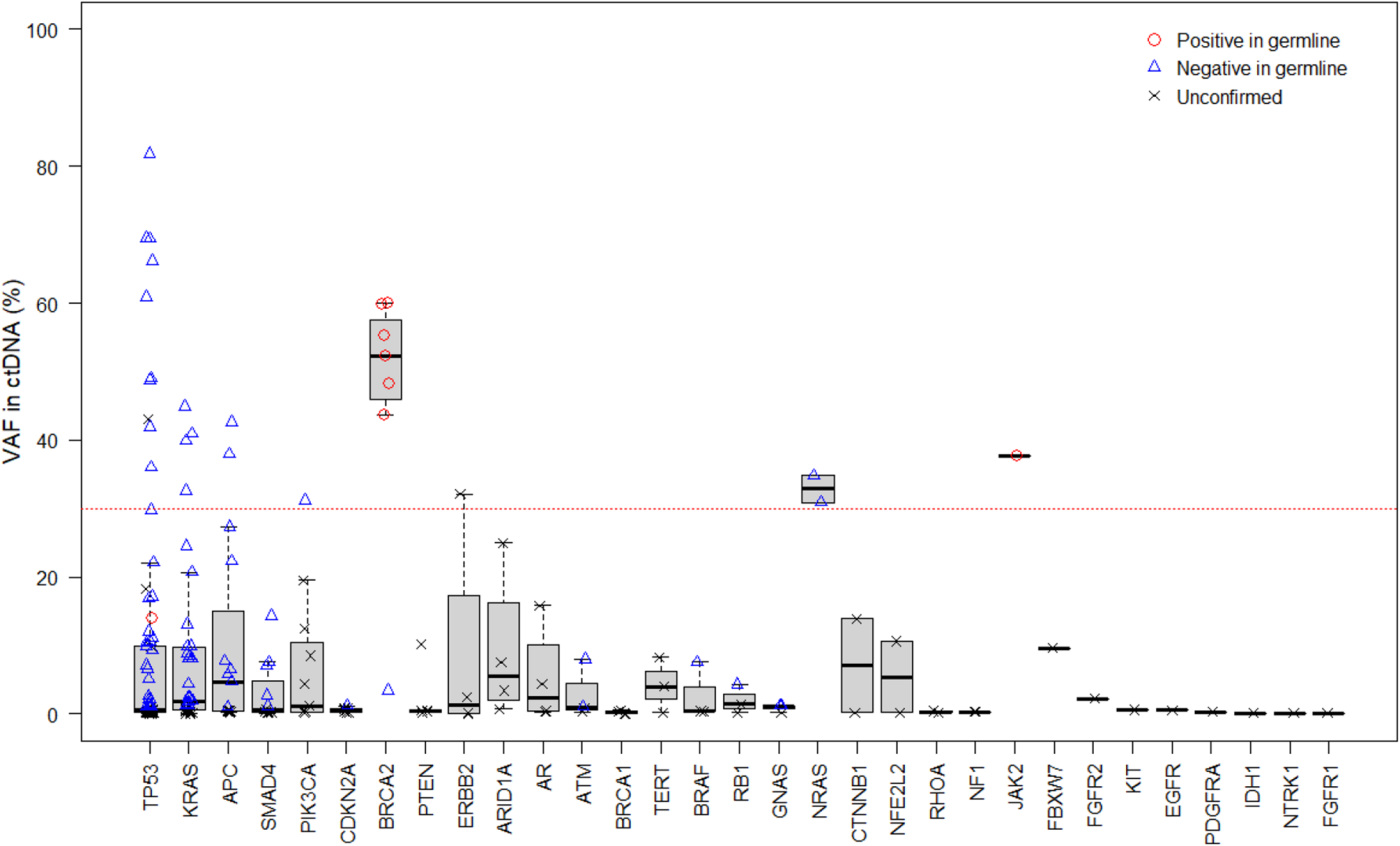
Distributions of variant allele fractions (VAFs) in ctDNA testing across genes. VAFs (%) in ctDNA testing are plotted by gene. Results of germline sequencing (positive, negative, or unconfirmed) are distinguished by symbols. The horizontal red dashed line indicates 30% VAF

**Fig. 4:**
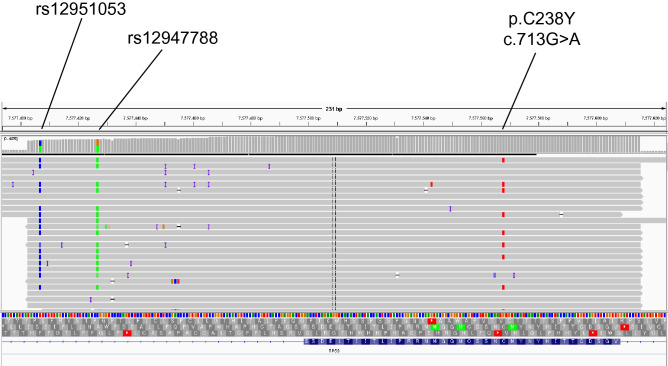
The distributions of the *TP53* C238Y variant and its common SNPs on the germline DNA sequencing reads. The *TP53* C238Y variant was detected in a fraction of sequencing reads originating from haploid genome harboring two neighboring heterozygous SNPs (rs12951053 and rs12947788) in the germline DNA sequencing

To confirm the origin of pathogenic variants, germline sequencing was performed using archived DNA from 44 patients whose ctDNA testing indicated pathogenic variants with ≥ 1% VAFs (Fig. 2). As a result, six *BRCA2* variants, which VAFs ranged from 43.7 to 60.0% in ctDNA testing, were confirmed to be germline in patients with pancreatic (*n* = 2), breast (*n* = 2), ovarian (*n* = 1), and prostate cancers (*n* = 1) (Fig. 3 and Table 2). In addition, germline sequencing illustrated possible acquired variants in *TP53* and *JAK2* genes (Fig. 3 and Table 2). VAFs of the *TP53* C238Y variant were 14.0 and 15.6% in ctDNA testing and germline sequencing, respectively (Table 2). In germline sequencing, no copy-number abnormality was detected in the *TP53* locus (Supplementary Figure S1). Further analysis of allelic read distribution in germline sequencing revealed that this variant was found only in a fraction of sequencing reads originating from haploid genome with two neighboring heterozygous SNPs (rs12951053, rs12947788) (Fig. 4). These results indicate that this variant was likely to be an acquired variant rather than a germline variant. VAFs of the *JAK2* V617F variant, commonly found in myeloproliferative neoplasm [19], were 37.7 and 46.1% in ctDNA testing and germline sequencing, respectively (Table 2). Since no heterozygous SNPs existed near this variant to compare the allelic distributions, we could not further clarify the origin of this variant.

**Table 2:** Variants detected in germline DNA sequencing

Cancer type	Age	Sex	Familial history of cancer	Gene	Coding	Amino acid	VAF ctDNA	VAF blood
Breast	47	Female	Prostate (f)	*BRCA2*	c.5645C > A	S1882*	52.2	49.1
Breast	70	Female	–	*BRCA2*	c.5576_5579delTTAA	I1859fs*3	53.6 (1st) 59.9 (2nd)	50.7
Prostate	57	Male	–	*BRCA2*	c.9212dupA	V3072fs*39	48.2	50.7
Pancreatic	46	Male	Breast (m), hepatic (f), head & neck (f)	*BRCA2*	c.9212dupA	V3072fs*39	55.2	49.9
Pancreatic	38	Female	Gastric (f), esophageal (f)	*BRCA2*	c.8504C > A	S2835*	43.7	52.9
Ovarian	44	Female	Prostate (gf)	*BRCA2*	c.6405_6509delCTTAA	N2135fs*3	60.0	50.4
Pancreatic	67	Male	Malignancy (f)	*JAK2*	c.1849G > T	V617F	37.7	46.1
Bile duct	58	Male	–	*TP53*	c.713G > A	C238Y	14.0	15.6

*f* father, *m* mother, *gf* grandfatheric

* indicates the termination codon

## Discussion

Since VAFs detected in ctDNA testing tend to be much lower (median VAF < 1%) than those in tissue-based testing [2, 3], variants with higher VAFs around 50% are expected to be of germline rather than somatic origin. The present study performed germline sequencing using peripheral blood DNA samples for pathogenic variants with ≥ 1% VAFs reported in ctDNA testing. Although peripheral blood DNA analysis found seven pathogenic variants out of 25 with ≥ 30% VAFs, only six were confirmed to be true pathogenic germline variants. In contrast, out of the 63 variants with VAFs ranging from 1 to < 30%, there were no true pathogenic germline variants. These results support the idea that a threshold of VAFs ≥ 30% is feasible to select presumed germline pathogenic variants, which require confirmatory germline testing in clinical ctDNA testing. A recent study reported that 89% of 36 confirmed germline variants exhibited ctDNA VAFs between 40 and 60%; the remaining 11% were out of this range [20]. Their report is consistent with our results; however, research also suggests that using a tight threshold of the ctDNA VAF range to screen germline variants can lead to missing true germline variants.

In the present study, we confirmed the presence of true pathogenic germline variants only in *BRCA2*, which could possibly be due to the following reasons: First, *BRCA2* was the most common cancer susceptibility gene in patients with advanced cancers who undergo universal germline sequencing [21]. Second, ctDNA panel (Guardant360^®^) utilized in this study did not cover several of the common cancer susceptibility genes, such as *CHEK2*, *MSH2*, *MSH6*, and *PMS2*. Third, the sample size was small, and over 50% of the patients had one of the top three cancer types (pancreatic, colorectal, and lung).

Variants of CH can contaminate the results of the ctDNA testing [22, 23]. CH is commonly observed in aged people and patients undergoing anticancer therapies [24, 25]. CH variants are found in various hereditary cancer-associated genes such as *TP53* and *ATM* [15, 23]. The VAFs for the *TP53* C238Y variant detected in this study were relatively low in ctDNA and peripheral blood DNA (14.0 and 15.6%, respectively), suggesting that this variant is not likely to be of true germline origin. Supporting this idea, the family history and clinical course of the patient did not meet the criteria for the Li-Fraumeni syndrome. Further analysis of allelic read distribution compared to common SNPs located nearby the *TP53* C238Y variant was helpful to confirm it is an acquired genetic variation. The *JAK2* V617F variant was detected in another patient with 46.1% VAFs in germline sequencing. This variant is a well-known somatic variant hotspot in myeloproliferative neoplasms and CH [19, 26], and this patient demonstrated a clinical manifestation of thrombocytosis. Although we could not obtain further information to identify its origin by germline sequencing, according to the patient’s phenotype, familial history, and known properties of this variant, we presumed that this was likely to be an acquired variant rather than a germline one. The recent publication of the ACMG statements suggested that analyzing additional control samples from other normal tissues from the patients or their family members would be helpful to distinguish the origin of variants [15].

This study exhibits the other limitation. Because little information was available about copy-number alterations from ctDNA testing, their effects on VAFs remain unknown in our germline analysis. Detailed information about copy-number alterations can improve the presumption accuracy of germline variants in ctDNA testing, as shown in previous reports [12, 27].

In conclusion, our current study demonstrated that VAFs information helps select putative germline variants in clinical ctDNA testing.

## Supplementary Information

Below is the link to the electronic supplementary material.

Supplementary file1 (PDF 47 KB)

Supplementary file2 (PDF 11 KB)
