# Supplementary material for: Prevalence of pathogenic germline variants in the circulating tumor DNA testing
Source: Int J Clin Oncol. 2022 Jul 23;27(10):1554–61. doi: 10.1007/s10147-022-02220-x (PMC9510107; doi:10.1007/s10147-022-02220-x)
Supplement: Supplementary file 1 — Supplementary file1 (PDF 47 KB) [file 10147_2022_2220_MOESM1_ESM.pdf]

Supplementary Figure S1

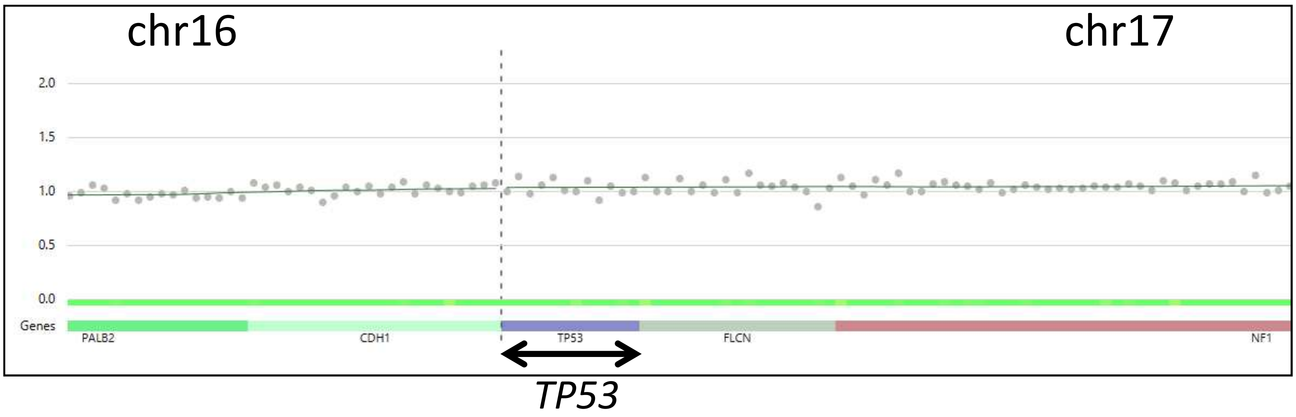

The result of copy number analysis in germline sequencing of the patient with *TP53* C238Y variant.
