# Supplementary material for: Prevalence of pathogenic germline variants in the circulating tumor DNA testing
Source: Int J Clin Oncol. 2022 Jul 23;27(10):1554–61. doi: 10.1007/s10147-022-02220-x (PMC9510107; doi:10.1007/s10147-022-02220-x)
Supplement: Supplementary file 2 — Supplementary file2 (PDF 11 KB) [file 10147_2022_2220_MOESM2_ESM.pdf]

Supplementary Table 1. Target genes in confirmatory germline sequencing.

Guardant360 target genes	covered by HCGP or CHP
AKT1	CHP
ALK	CHP
APC	HCGP, CHP
ATM	HCGP, CHP
BRAF	CHP
BRCA1	HCGP
BRCA2	HCGP
CDH1	HCGP, CHP
CDK4	HCGP
CDKN2A	HCGP, CHP
CTNNB1	CHP
EGFR	CHP
ERBB2	CHP
EZH2	CHP
FBXW7	CHP
FGFR1	CHP
FGFR2	CHP
FGFR3	CHP
GNA11	CHP
GNAQ	CHP
GNAS	CHP
HNF1A	CHP
HRAS	CHP
IDH1	CHP
JAK2	CHP
JAK3	CHP
KIT	CHP
KRAS	CHP
MET	HCGP, CHP
MLH1	HCGP, CHP
MPL	CHP
NF1	HCGP
NOTCH1	CHP
NPM1	CHP
NRAS	CHP
PDGFRA	CHP
PIK3CA	CHP
PTEN	HCGP, CHP
PTPN11	CHP
RB1	HCGP, CHP
RET	HCGP, CHP
SMAD4	HCGP, CHP
SMO	CHP
STK11	HCGP, CHP
TP53	HCGP, CHP
TSC1	HCGP
VHL	HCGP, CHP

Abbreviations: HCGP, hereditary cancer-associated gene panel; CHP, Ion AmpliSeq cancer hotspot panel v2
